# Hybrid phenotype mining method for investigating off-target protein and underlying side effects of anti-tumor immunotherapy

**DOI:** 10.1186/s12911-020-1105-4

**Published:** 2020-07-09

**Authors:** Yuyu Zheng, Xiangyu Meng, Pierre Zweigenbaum, Lingling Chen, Jingbo Xia

**Affiliations:** 1 Hubei Key Lab of Agricultural Bioinformatics, College of Informatics, Huazhong Agricultural University, Wuhan, 430070 China; 2 Department of Urology, Zhongnan Hospital of Wuhan University, Wuhan, China; 3 Institut Curie, CNRS, UMR144, Molecular Oncology Team, PSL Research University, Paris, France; 4 Université Paris-Saclay, CNRS, LIMSI, Orsay, France

**Keywords:** Immune checkpoint, PD-1, PD-L1, off-target effect, CRF

## Abstract

**Background:**

It is of utmost importance to investigate novel therapies for cancer, as it is a major cause of death. In recent years, immunotherapies, especially those against immune checkpoints, have been developed and brought significant improvement in cancer management. However, on the other hand, immune checkpoints blockade (ICB) by monoclonal antiboties may cause common and severe adverse reactions (ADRs), the cause of which remains largely undetermined. We hypothesize that ICB-agents may induce adverse reactions through off-target protein interactions, similar to the ADR-causing off-target effects of small molecules. In this study, we propose a hybrid phenotype mining approach which integrates molecular level information and provides new mechanistic insights for ICB-associated ADRs.

**Methods:**

We trained a conditional random fields model on the TAC 2017 benchmark training data, then used it to extract all drug-centric phenotypes for the five anti-PD-1/PD-L1 drugs from the drug labels of the DailyMed database. Proteins with structure similar to the drugs were obtained by using BlastP, and the gene targets of drugs were obtained from the STRING database. The target-centric phenotypes were extracted from the human phenotype ontology database. Finally, a screening module was designed to investigate off-target proteins, by making use of gene ontology analysis and pathway analysis.

**Results:**

Eventually, through the cross-analysis of the drug and target gene phenotypes, the off-target effect caused by the mutation of gene BTK was found, and the candidate side-effect off-target site was analyzed.

**Conclusions:**

This research provided a hybrid method of biomedical natural language processing and bioinformatics to investigate the off-target-based mechanism of ICB treatment. The method can also be applied for the investigation of ADRs related to other large molecule drugs.

## Background

Immunotherapy has been very popular in cancer treatment recently, owing to the breakthroughs made in the past few decades, including investigation of mechanisms and related immune response [1]. Mechanisms of action vary among the different types of cancer immunotherapies including oncolytic viruses therapy, cancer vaccines and monoclonal antibodies therapy. Unlike the traditional immunotherapies, the monoclonal antibodies therapy accurately targets an inhibitory-signaling pathway, which is related to Programmed Death-1/Programmed cell Death-Ligand 1 (PD-1/PD-L1) [2]. Monoclonal antibodies are proteins produced by immune cells that specifically recognize cellular targets in the context of cancer treatment. Monoclonal antibodies can inhibit the activity of specific proteins in cancer cells to kill cells or prevent them from growing [3].

Immunotherapy has cured many people’s immune-related diseases, brought new medical tools to humans, and promoted new developments in medicine. However, while curing the disease, it also brings many side-effect diseases to people. Therefore, studying the side-effect mechanism of immunomonoclonal drugs can better provide more powerful evidence for drug modification and production.

### PD-1/PD-L1 mechanism of action

The binding of PD-1 to its ligand PD-L1 is critical for the homeostatic regulation of the immune system. The primary role of the PD-1/PD-L1 signaling process is to suppress autonomic T cells and accelerate programmed cell death of T cells for the prevention of human immune diseases. After antigen receptor and cytokine signaling, PD-1 is expressed on mature CD4+ and CD8+ T cells, killer T cells, and some cell subsets of B cells [4]. PD-L1 is ubiquitously expressed in a wide variety of immune and tumor cells, such as T cells, B cells, macrophages, regulatory T cells, dendritic cells, and some non-immune cell types, such as vascular endothelial cells and pancreatic cells [5]. However, in the tumor microenvironment, PD-L1 levels in bone marrow cells (eg, macrophages, MDSCs, and DCs) are much higher than in tumors and stromal cells, compared to much lower PD-L1 expression in lymphocytes [6].

**Fig. 1:**
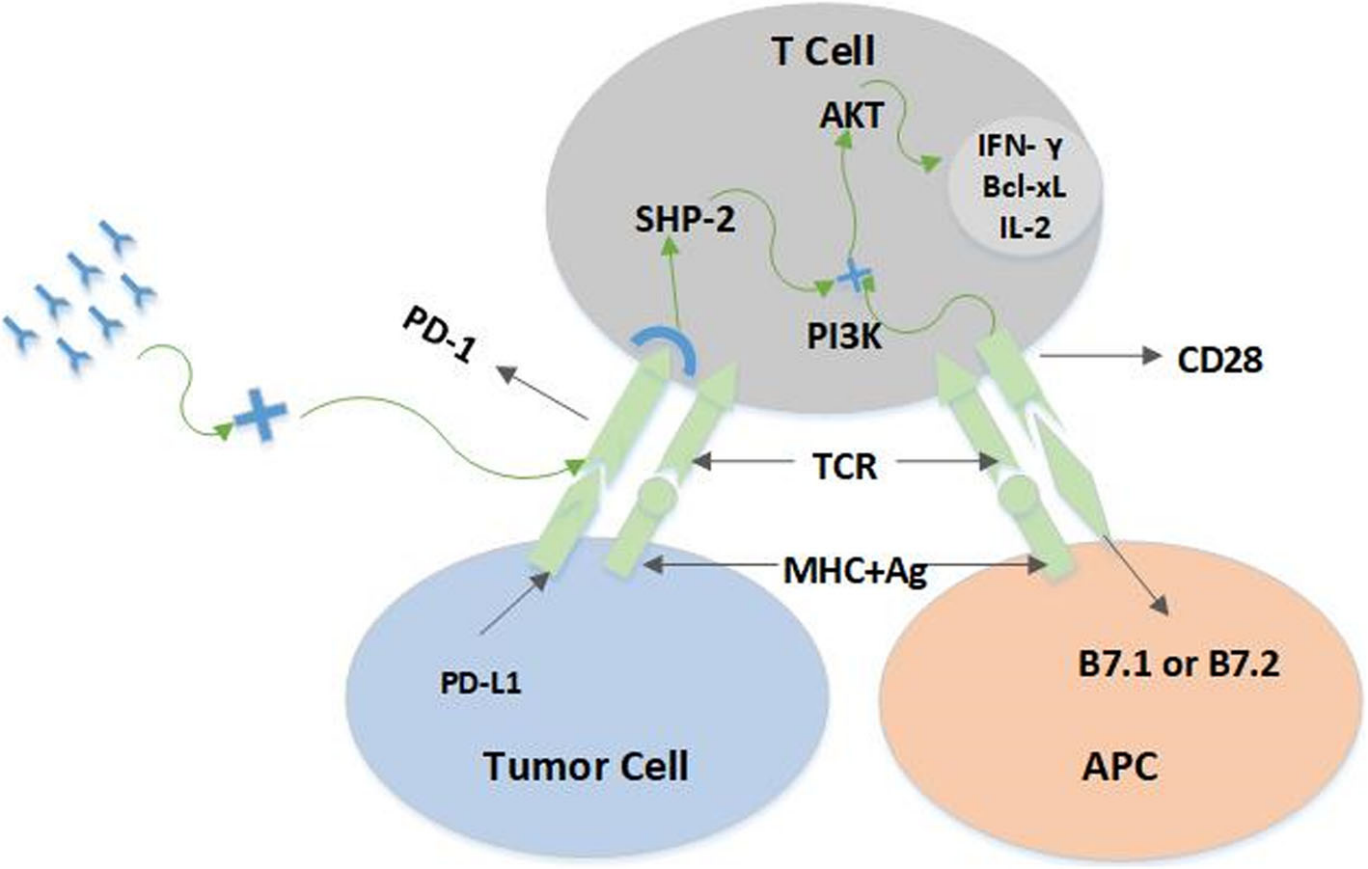
PD-1/PD-L1 immune pathway map. The figure shows the amino acid sequences of the heavy and light chains of Pembrolizumab

In a tumor-free environment, PD-L1 expression is restricted in normal tissues [7]. Binding of B7.1 or B7.2 on APC to CD28 on T cells upregulates T cell survival protein (IFN- *γ*, Bcl-xL and IL-2) expression by PI3K activation of T cell intracellular AKT pathway. The T cell proliferation initiates an anti-tumor response, as shown in Fig. 1. In contrast, in the tumor environment, PD-L1 expressed on tumor cells binds to PD-1 located on T cells and is enriched by phosphatase SHP-2. The AKT pathway causes T cell survival protein expression to decrease, resulting in T cell immune failure, as shown in Fig. 1 [8]. However, in recent studies, it has been found that most of the “contribution” in the process of tumor escape is derived from the host’s own cells. In this case, PD-L1 is not expressed in most of the tumor cells, but highly expressed in the adjacent cells around the tumor cells and inflammatory cells. It is reported that PD-L1, which is expressed at high levels on bone marrow cells, plays a important role in suppressing T cell responses [6]. When a greater inhibitory effect is activated in tumor cell, the expression of PD-L1 on tumor cells shows transient and diminishes rapidly. If the tumor is in an early stage and has just begun to evade immune surveillance, its effect is stronger [9]. For example, the relative importance of tumor PD-L1 may be affected by the inherent immunogenicity of the tumor. If the immune prototype is strong, the tumor’s PD-L1 is sufficient to trigger immune escape [10]. In addition, the degree of expression of PD-L1 is related to other genetic variations. After the epidermal growth factor receptor is mutated into NSCLC cells, PD-L1 is biased to be expressed on the cell surface [5].

The PD-1/PD-L1 inhibitor targets the epitope on the PD-1/PD-L1 molecule with high affinity and specificity. By blocking their interaction pathways, T cells can “recognize” cancer cells and initiate immune responses [11].

### Side effect and phenotype mining

The PD-1/PD-L1 inhibitor drugs that have been approved by the FDA are mainly: 1. Anti-PD-1 antibodies: Nivolumab developed by BMS, Pembrolizumab of Merck, 2. Anti-PD-L1 antibody: Atezolizumab, Pfizer of Roche /Avelumab from Merck, Germany, Durvalumab from AstraZeneca, etc [12, 13] (See Table 1). Side effects of drugs are related to adverse reactions in human medicine, and it is regarded as a typical phenotype. The common side effects of PD-1 drugs include constipation, fever, cough, dyspnea, headache, hair loss, chills, rapid heartbeat, sore throat, abnormal fatigue or muscle weakness, sputum and stiffness, joint pain, muscle cramps and stiffness, hoarseness, abnormal weight [14]. Common side effects of PD-L1 drugs include: bladder pain, fever, cough, urinary blood stasis or turbidity, difficulty urinating, burning or pain, numbness of hands and feet, watery or bloody diarrhea, hoarseness, abnormal weight, chills, stomach cramps, extremely tired or weak, waist pain, rapid or slow heartbeat, swelling of the face or limbs [15].

**Table 1:** Five drugs against PD-1/PD-L1

Drug	Trademark	Owner	Drug target	Approval date
Pembrolizumab	Keytruda	MSD	PD-1	Sep 2014
Nivolumab	Opdivo	BMS	PD-1	Dec 2014
Atezolizumab	Tecentriq	Roche	PD-L1	May 2016
Avelumab	Bevancio	EMD and Pfizer	PD-L1	Mar 2017
Durmalumab	Imfinzi	Astra Zeneca	PD-L1	May 2017

Campillos et al. [16] suggested that if there is an identical side-effect phenotype between different drugs, it reveals that these drugs share a target. Their results show that analyzing the side-effects of drugs can reveal new targets for drug action. Actually, novel targets offer new clinical treatment possibilities. Keiser et al. [17] used a statistical-based chemical informatics approach to predict new off-targets for drug compounds. The similarity ensemble approach (SEA)was used to calculate the structural similarity between drugs and targets, and to infer drug-target associations.

Furthermore, using the adverse effects of drug targets to establish a large-scale drug-target-adverse reaction network is a novel way to explain the ADR coincidence between target genes and drug target genes [18, 19]. Owing to the rapid growth of biomedical literature in recent years, it is feasible to automatically extract knowledge from the published papers, such as the drugs and diseases they mention and their relations in a large scale [20, 21]. The knowledge found can be used in many biomedical related fields such as drug discovery, safety monitoring, and drug side-effect detection [22, 23]. Manually identifying such entities can be very accurate, but is also time consuming and laborious. Developing automatic annotation systems based on Natural Language Processing (NLP) technology is an alternate method to identify drug and disease entities in text [24]. It may be applied in large scale and provide a way to discover relations among phenotypes and off target effects.

In this paper, a pharmacological knowledge mining strategy iss proposed in the form of hybrid phenotypic mining by combining Biomedical Natural Language Processing (BioNLP) with medical informatics. First, we targeted at five PD-1/PD-L1 inhibitor drugs, and extract all the adverse drug reaction (ADR) from DailyMed drug labes by using conditional random fields (CRF) [19]. Second, we targeted at all the targeted proteins of the drugs, and extracted all the phenotype terms of the proteins from the Human Phenotype Ontology (HPO). To integrate the phenotype from ADR and HPO terms, we cross matched the phenotype terms with embedding similarity and find intersected terms, which was eventually used to investigate the target phenotype. As a result, the mutation of gene BTK was investigated and found to be relevant to the off-target effect of anti-tumor immunology therapy.

## Material and method

### Materials

#### Drugs and drug labels

Five drugs against PD-1/PD-L1 are listed at Table 1, and their amino acid sequences were collected from Drugbank [12, 13].

The N-terminal of an lgG antibody, approximately 120 amino acids, is a complementarity determining region (CDR). It was recently found that the heavy chain CDR2 of Avelumab binds to a specific part of PD-L1 and prevents PD-1 from being linked to PD-L1, thereby blocking the channel effect of PD-1/PD-L1 [25]. Both Atezolizumab and Durvalumab use all three CDRs from the heavy chain and two complementarity determining regions from the light chain to form contact with PD-L1 [26]. All three CDR loops in the Nivolumab VH region and the CDR1/CDR2 loops in VL provide partial interaction, without contact of LCDR3. Nivolumab and Pembrolizumab bind to different parts of PD-1, and these parts overlap, preventing Pembrolizumab antibodies from continuing to bind to PD-1 [27]. Therefore, the CDR region sequence of the light and heavy chain of the drug protein sequence was used in this research for BlastP alignment, which consisted of ∼240 amino acids.

**Fig. 2:**
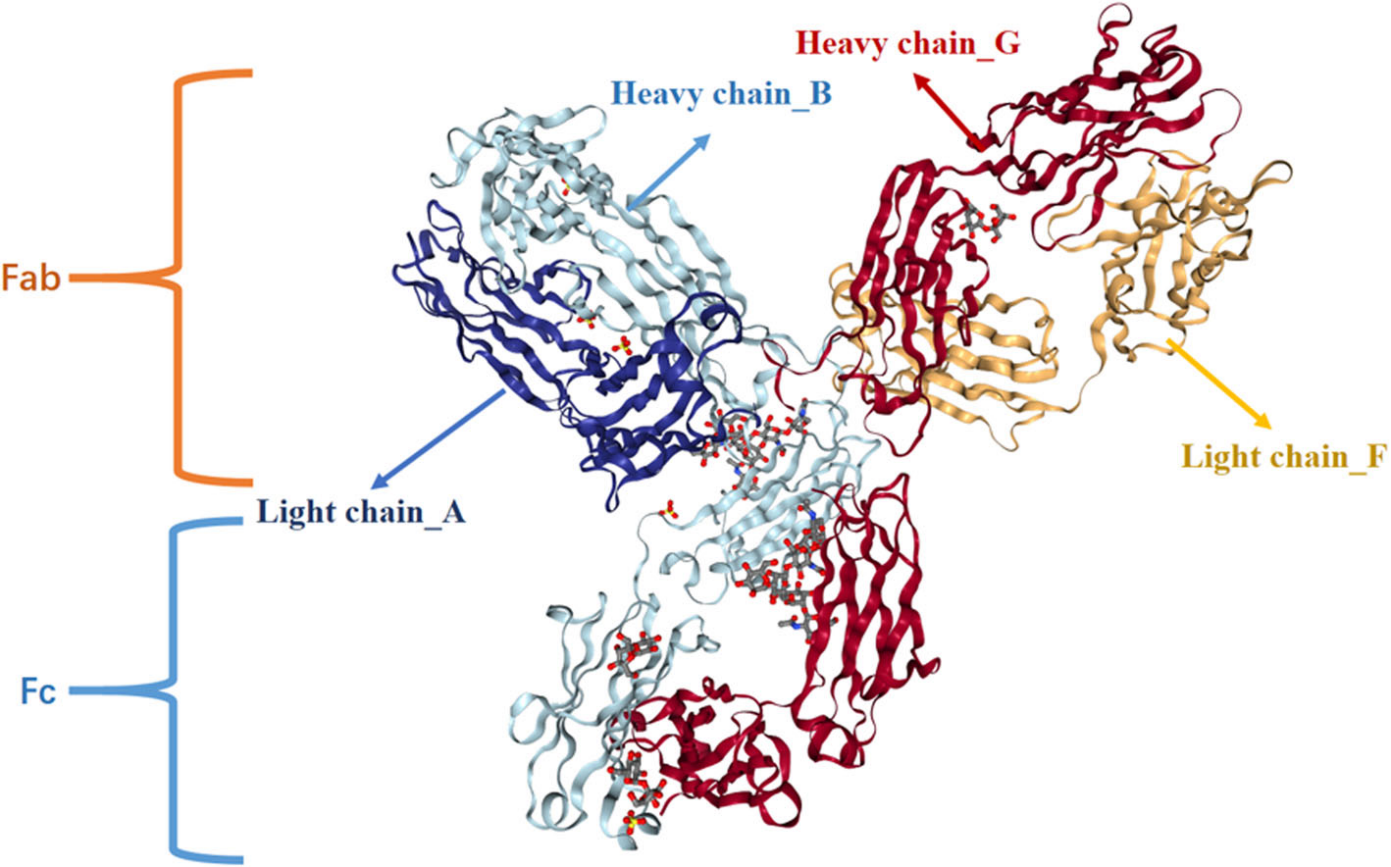
Crystal Structure of Pembrolizumab. The figure shows the amino acid sequences of the heavy and light chains of Pembrolizumab.The antibody Pembrolizumab is divided into two parts, which are the Fab and Fc segments in the figure. The four arrows indicate different chains

Taking Pembrolizumab as an example, the amino acid sequences of the heavy and light chains are shown in the Fig. 2. The antibody consists of 4 strands, and the Fab segment is the part that can specifically recognize the antigen.

Heavy Chain Sequence QVQLVQSGVEVKKPGASVKVSCKASGYTFTNYYMYWVRQAPGQGLEWMGGI NPSNGGTNFNEKFKNRVTLTTDSSTTTAYMEL

KSLQFDDTAVYYCARRDYRFDMGFDYWGQGTTVTVSSASTKGPSVFPLAPC SRSTSESTAALGCLVKDYFPEPVTVSWNSGAL

TSGVHTFPAVLQSSGLYSLSSVVTVPSSSLGTKTYTCNVDHKPSNTKVDKR VESKYGPPCPPCPAPEFLGGPSVFLFPPKPKD

TLMISRTPEVTCVVVDVSQEDPEVQFNWYVDGVNAKTKPREEQFNSTYRVV SVLTVLHQDWLNGKEYKCKVSNKGLPSSIEKT

ISKAKGQPREPQVYTLPPSQEEMTKNQVSLTCLVKGFYPSDIAVEWESNGQ PENNYKTTPPVLDSDGSFFLYSRLTVDKSRWQ

EGNVFSCSVMHEALHNHYTQKSLSLSLGK

Light Chain Sequence

EIVLTQSPATLSLSPGERATLSCRASKGVSTSGYSYLHWYQQKPGQAPRLL IYLASYLESGVPARFSGSGSGTDFTLTISSLE

PEDFAVYYCQHSRDLPLTFGGGTKVEIKRTVAAPSVFIFPPSDEQLKSGTA SVVCLLNNFYPREAKVQWKVDNALQSGNSQES

VTEQDSKDSTYSLSSTLTLSKADYEKHKVYACEVTHQGLSSPVTKSFNRGEC

As training data for phenotype extraction, we downloaded the gold dataset of the TAC 2017 shared task on adverse reaction extraction [28], which consists of 100 drug labels and annotated adverse reactions. Meanwhile, the drug labels of 5 targeted drugs were downloaded from DailyMed [29] (https://dailymed.nlm.nih.gov/dailymed/) in XML format.

#### Omics data sets

Several multi-Omics data sets were used in this research.

i) HPO. Human phenotype ontology (HPO) contains standardized vocabulary for phenotypic abnormalities in human diseases [30].
ii) UniproKB. We used the local BlastP [31] and protein database UniprotKB [32] to obtain homologous sequences of five drugs.
iii) STRING. Protein-protein interaction (PPI) analysis helps to study the molecular mechanisms of disease and discover new drug targets. In this paper, antibody-producing proteins may be obtained from the STRING database.

### Proposed method: hybrid phenotype mining method

Though the exact physiological process of immune-related side effects is unclear, it is thought to be closely related to maintaining immune homeostasis. Since most immune-related adverse reactions resolve within weeks to months after the onset of immunosuppressive therapy, one of the most important clinical practice is to restore the safety of immune checkpoint blockade after the adverse events have subsided. Because the study protocol often requires that if there is a serious adverse reaction related to the immune system, the immune checkpoint blockade treatment must be permanently stopped, so the prospective data from clinical trials is limited [33]. Therefore, a deeper understanding of these mechanisms will help us find new ways to effectively deal with PD-1/PD-L1 tumor escape and solve the above safety problems.

**Fig. 3:**
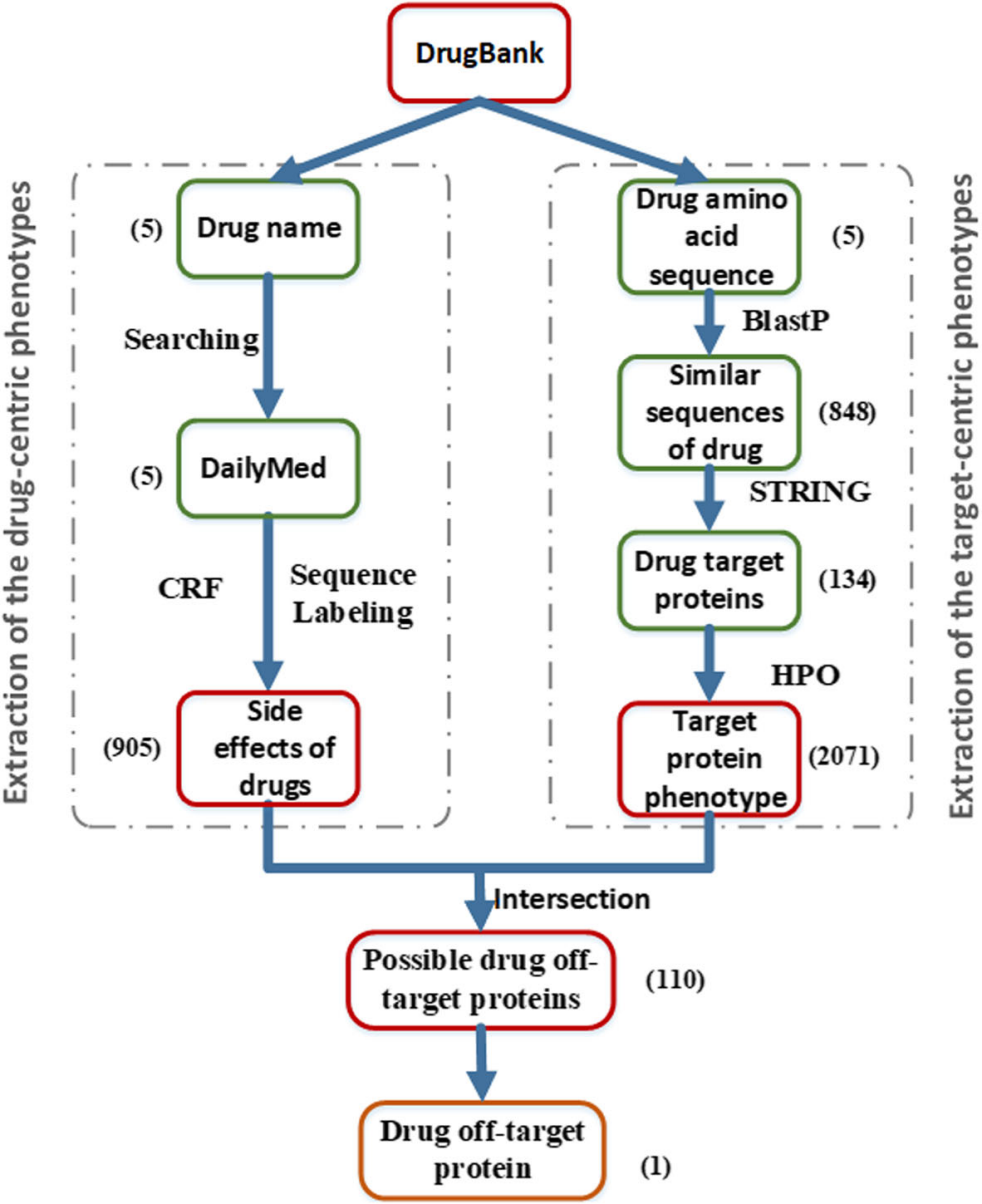
Flow chart of the hybrid phenotype mining method. The figure shows the general flow chart of the proposed hybrid phenotype extraction method.See the article for specific procedures

Figure 3 shows the general flow chart of the proposed hybrid phenotype extraction method. The pipeline consists of one pre-processing step and three computation modules, i.e., extraction module of drug-centric phenotypes, extraction module of target-centric phenotypes, screening module for off-target proteins.

**Pre-processing module.** This module downloads the anti-PD-1/PD-L1 drug from the Drugbank database, and the XML description file from the DailyMed database. In addition, it downloads the protein sequence of the antibody drug from the Drugbank database.

**Extraction module of drug-centric phenotypes.** As shown in the left module section in Fig. 3, drug-centric phenotype terms were extracted from DailyMed texts of drug labels with Conditional Random Fields. Wapiti [34] is used as the CRF implementation.

- **Step-**1: **Formatting drug label texts.** The BIEO scheme is used as the entity tagging format. ADRs in the results are marked as B-AdverseReaction, I-AdverseReaction, E-AdverseReaction, where B-type means begin, I-type means Inside, E-type means End, and O indicates Outside. For instance, if the word “pruritis” is an ADR entity, it is marked as “B-AdverseReaction”; if a phrase “injection site hemorrhage” is an ADR entity, the phrase is marked as “B-AdverseReaction I-AdverseReaction E-AdverseReaction”.
- **Step-**2: **Setting up feature function.** DISO (Disorders) is a semantic group in the UMLS [35] through which we built a dictionary of disorder terms from the UMLS Metathesaurus [36]. This semantic group consists of the following 12 semantic types: acquired abnormality, anatomical abnormality, cell or molecular dysfunction, congenital abnormality, disease or syndrome, experimental model of disease, finding, injury or poisoning, mental or behavioral dysfunction, neoplastic process, pathologic function, and sign or symptom.
- **Step-**3: **NER of phenotype terms with CRF.** Gold standard annotated text from the TAC shared task is first used to learn a CRF model. That model is then applied to the unannotated DailyMed drug label texts to detect drug side effects. The training data is randomly divided according to 7: 3. During the training model, 7 copies use the actual training model and 3 copies use the development set to adjust the parameters. After the model is optimized, all data is applied to the DailyMed drug label texts to detect side effect mentions.

**Extraction module of target-centric phenotypes**. As shown in the right module section in Fig. 3, target-centric phenotype terms were extracted from a series of database queries, including BlastP, STRING, and HPO.

- **Step-**1: **Sequence similarity computation.** We used the native BlastP and protein database UniprotKB to obtain homologous sequences of the five drugs. The parameters were set to: Indentities >30*%* and E-value >10−*e*4.
- **Step-**2: **PPI extension for searching target with function relevance.** We entered the protein name in the BlastP result into the STRING database, setting the confidence parameter to 0.7 and the maximum number of display nodes to 500.
- **Step-**3: **Target-centric phenotypes extraction with HPO query.** We downloaded the HPO database and compared the previous protein interaction results with the HPO database. We thus obtained the side effect phenotype of each drug, and predicted the most likely drug off-target site.

**Screening module for off-target proteins**. A Gene Ontology (GO) analysis is performed using the obtained gene list information. We analyzed the signal pathways involved in each gene to screen for genes related to T cell proliferation activation, immune regulation, such as AKT and PI3K pathways, and matched the genes by cross-checking. Eventually, we performed literature search to find evidence supporting their relevance.

## Results

In this section, the results of the proposed hybrid phenotype mining method are given. Specifically, drug- and target-centric phenotype mining results were obtained and comprehensively integrated to unveil a cluster of possible target proteins of five drugs.

### Results for drug-centric phenotypes extraction

The precision (proportion of correctly predicted entities), recall (proportion of gold entities actually predicted) and F-score (harmonic mean of precision and recall) obtained on the training data for each entity type are shown in Table 2.

**Table 2:** CRF Model Optimization Result for Entity Detection

	Precision	Recall	F-Score	Number of occurrences
ADR	88.05%	68.30%	76.93	2,469
Animal	73.33%	50.00%	59.46	15
DrugClass	0.00%	0.00%	0.00	1
Factor	82.69%	33.33%	47.51	52
Negation	57.14%	28.57%	38.10	7
Severity	73.55%	29.87%	42.48	121

**Fig. 4:**
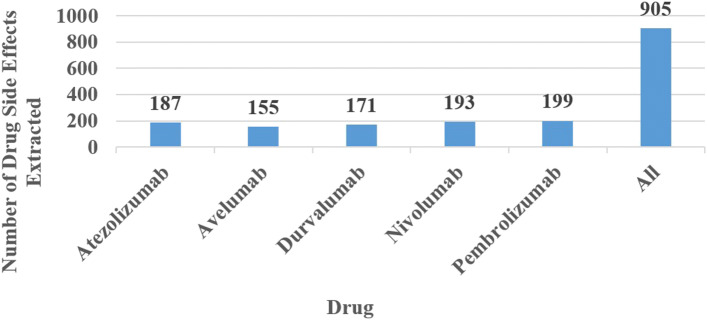
Number of Drug Side Effects Extracted. This figure shows the number of side effects of the extracted drug. The horizontal axis is 5 drugs, and the vertical axis is the number of side effects

After training, the model was used to extract all adverse reactions from the drug labels of the five targeted drugs. The extraction results are shown in Fig. 4. As can be seen from the figure, many side effects are extracted for each drug. An examination of the results shows many synonyms, which need to be further screened. A total of 905 drug side effects were extracted from the five drug labels.

### Results for target-centric phenotypes extraction

**Fig. 5:**
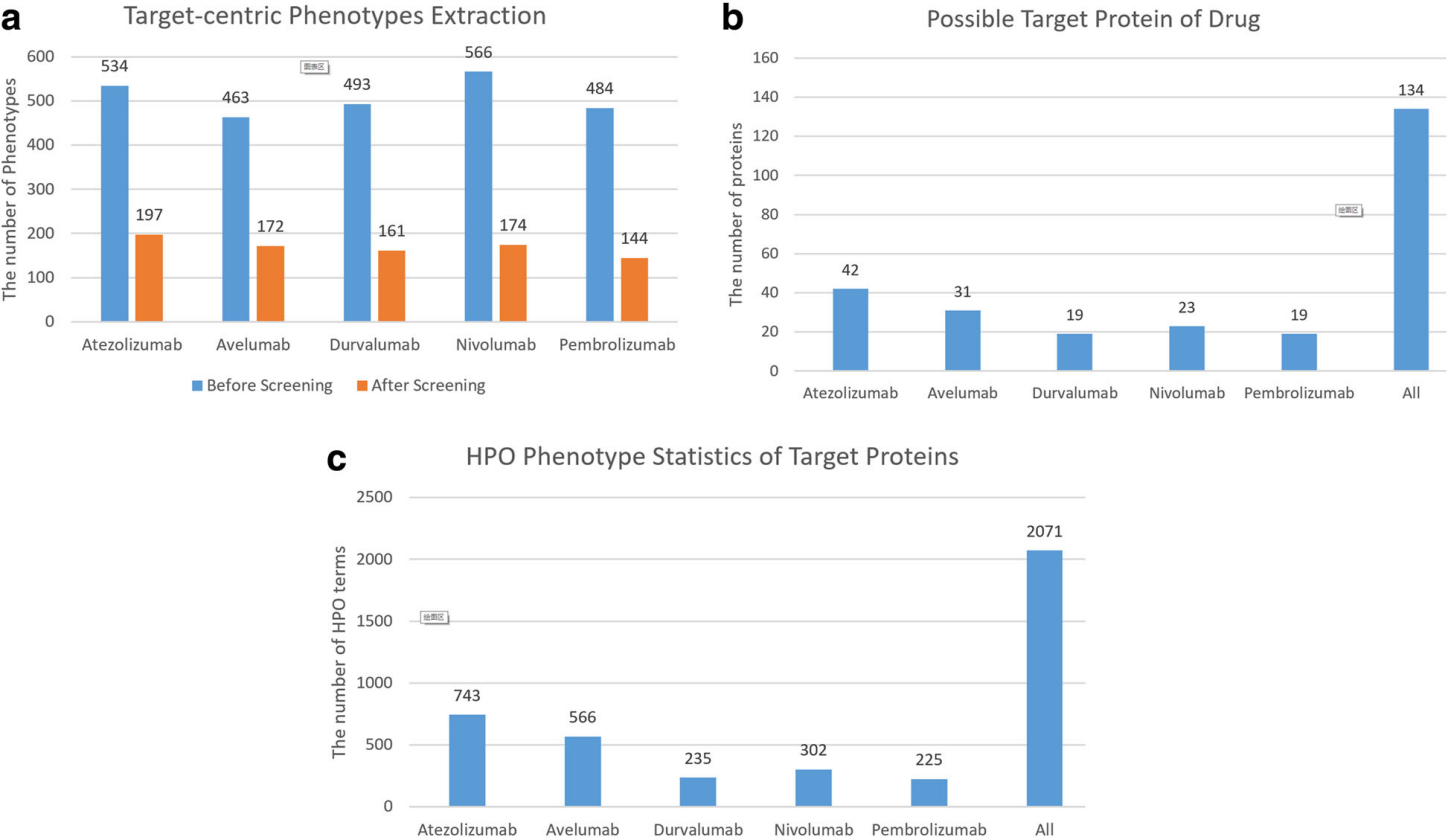
Target-centric Phenotypes Extraction. **a** Target-centric Phenotypes Extraction.The figure shows the number of side-effect phenotype changes before and after screening. **b** Possible Target Protein of Drug.The figure shows the number of possible side effects target proteins for the corresponding drug. **c** HPO Phenotype Statistics of Target Proteins

The results for the target-centric phenotypes are shown in Fig. 5.

i) **BlastP results**. We run BlastP to obtain proteins with similar structures of five target drugs. In this process, a total of 2,540 protein sequences were found before screening, and 848 sequences remained after screening. In the pre-screening results, some proteins will have different positions to match the drug protein sequence, so the number of proteins before screening will be repeated. For instance, P0DOX2, P0DOX3, P0DOX4, P0DOX5, P0DOX6, P0DOX7, P0DOX8 are immune one strand of globulin that do not have its corresponding gene name, so the number of proteins after screening is reduced. Although the five drugs were reduced before and after screening, the reduction trend was about the same, with Pembrolizumab decreasing the most, i.e., 70%. The statistic summary of the protein after BlastP is shown in Fig. 5a.
ii) **STRING results**. We use STRING to obtain proteins that have similar structure with BlastP screened proteins. Thus an expanded candidate off-target protein set is obtained. The statistic summary is shown in Fig. 5b. As can be seen from the figure, a total of 134 possible target proteins were found, of which Atezolizumab found 31%, while Pembrolizumab found fewer proteins, i.e., only 14%.
iii) **HPO results**. HPO terms were extracted from the drug labels of the five targeted drugs. As can be seen from Fig. 5c, a total of 2,071 HPO terms were found, of which Atezolizumab accounted for the most, at 36%, and Pembrolizumab had the least proportion, at 11%.

### Results for off-target proteins screening

**Fig. 6:**
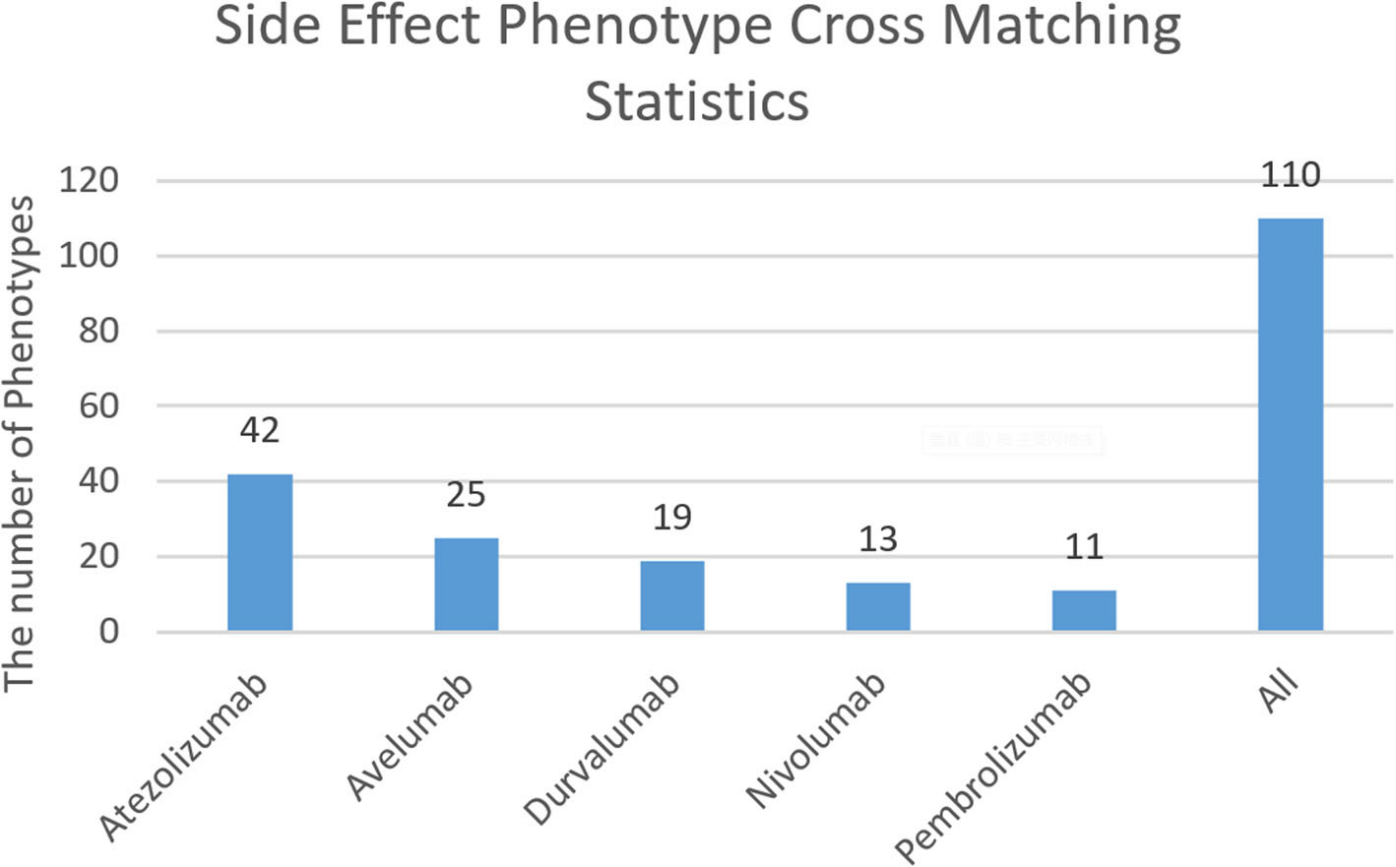
Side Effect Phenotype Cross Matching Statistics. The ordinate in the figure is the number of the adverse reaction terms in the drug label that intersect with the HPO terms in the candidate off-target protein
ii) **GO analysis results**. Gene ontology analysis aims to investigate the function of the filtered proteins. By filtering the genes with function in the signal pathways related to T cell proliferation activation, immune regulation, only three genes remains, which are AKT1, ACTG2, and BTK. The results of drug/gene/phenotype link are shown in Table 3.

i) **Side effect phenotype cross matching results**. We use embedding similarity strategy [19] to match intersected terms in adverse reaction terms from drug labels and HPO terms from candidate off target proteins. As can be seen from Fig. 6, the total match has 110 side effects, of which Atezolizumab has the largest proportion of 38%, and Pembrolizumab has the least proportion of 10%.

**Table 3:** Gene Ontology Analysis Results

Drug	Side Effect	Related Genes
Atezolizumab	Sepsis	ACTG2
Atezolizumab	Hyperthyroidism	AKT1
Avelumab	Diarrhea	ACTG2
Avelumab	Hyperthyroidism	AKT1
Avelumab	Cellulitis	BTK
Durvalumab	Sepsis	ACTG2
Nivolumab	Diarrhea	ACTG2
Pembrolizumab	Diarrhea	ACTG2
Pembrolizumab	Cellulitis	BTK
  In the result of GO analysis, ACTG2 is closely related to T cell activation and immune process, and may be associated with drug off-target. AKT1 is associated with inflammatory factor-mediated diseases, in which IFN- *γ* is also involved in immunosuppressive signaling, helping tumors to escape. Bruton’s tyrosine kinase (BTK) is involved in the activation process of B cells and is involved in immune regulation. However, the other two genes did not find relevant side effects, and there may be few articles reporting the corresponding side effects of the gene, so no relevant literature was found.

## Discussion and conclusion

In this section, the process of phenotype mining is discussed, and a candidate side-effect off-target site is proposed and analyzed accordingly.

### Phenotypic mining strategy on finding BTK

The screening process of BTK discovery was represented in Fig. 3 with respect to the numbers of hit counts for drugs, phenotypes and candidate core proteins.

First, the module of drug-centric phenotype extraction started with five PD-1/PD-L1 inhibitors, searched the DailyMed database for related side-effect phenotypes, and used a CRF for sequence labeling to mine 905 drug-centric side effects. The precision of that process was 88% on the training data, so a moderate amount of noise is expected. This noise should however be reduced by the intersection with target-centric phenotype extraction. Conversely, recall was 68%, meaning that about one third of the drug side effects are likely to be missed.

Second, in the module of target-centric phenotype extraction, a total of 848 amino acid sequences were found to be similar with sequences of the five PD-1/PD-L1 inhibitors by using blastP. Subsequently, a total of 134 target proteins of drug were found in the STRING database. Finally, the 134 proteins were compared with the HPO database, and a total of 2,071 target-centric side-effect phenotypes were found.

Third, in the screening module for off-target proteins, the phenotypes of the first two steps were cross-matched, and a total of 110 target-centered target proteins were found. For more accurate screening, we performed GO analysis and found genes related to AKT and PI3K pathways. A total of three were found: ACTG2, AKT1 and BTK. Since only BTK related literature reports were found during the literature search, only one gene related to PD-1/PD-L1 off-target was found.

### Candidate side-effect off-target site

As introduced in the result section, the proposed hybrid phenotype extraction method found a related side-effect off-target site, BTK, and we hypothesized that the off-target site caused a cellulitis side effect due to mutation of BTK gene.

While tracing back the side effect of Avelumab and Pembrolizumab in Table 3, both of which being relevant with BTK and suffered cellulitis, it is possibly BTK that played a role of side-eefect off-target site in terms with PD-1/PD-L1 blockage.

**Fig. 7:**
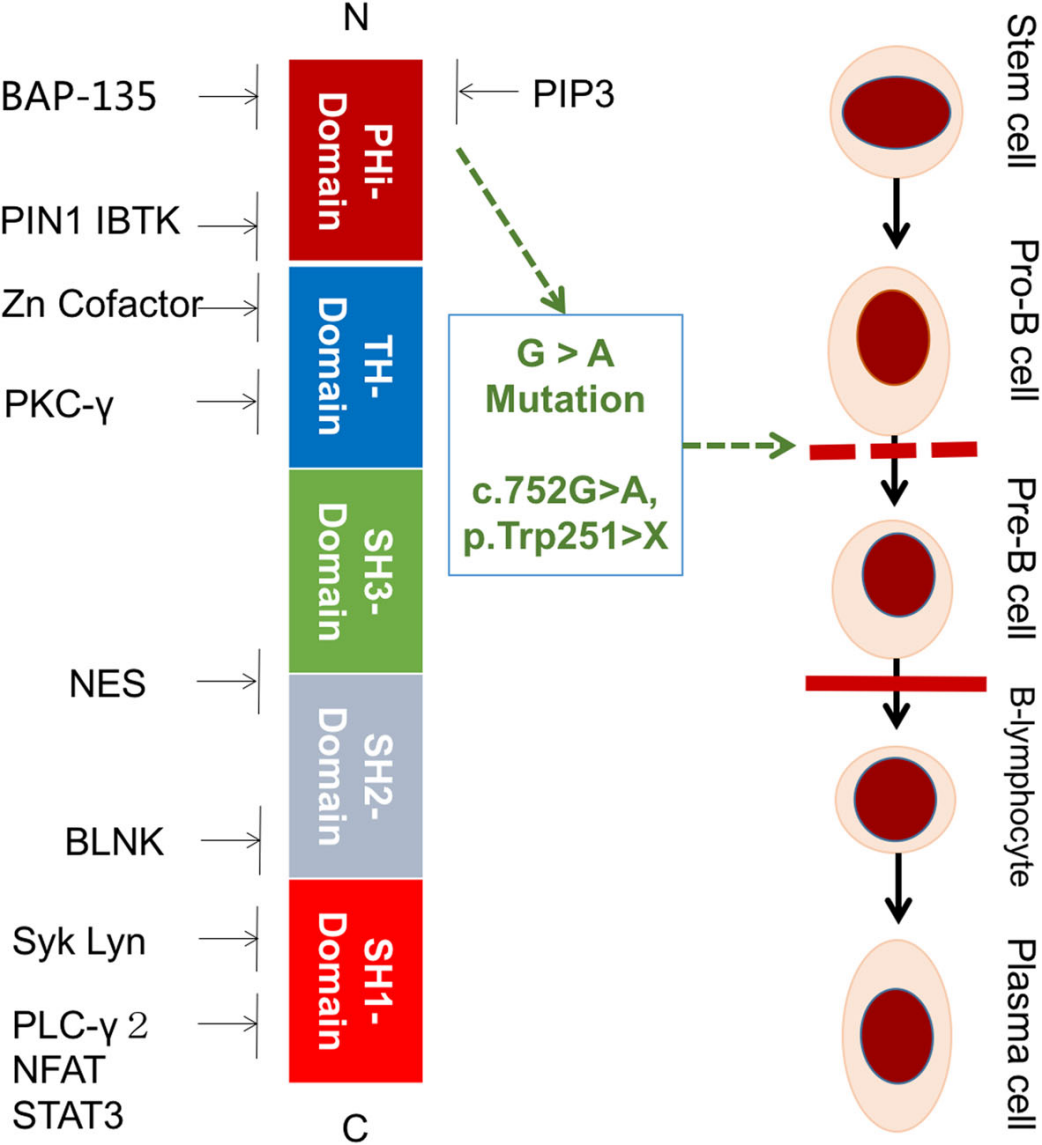
The domain strucuture of BTK, and the fact of B lymphocyte inhibition due to BTK mutation

Literature research unveiled the fact that BTK is associated with B cell activation. If B cells are not activated, the efficiency of immunization is greatly reduced, and it also affects the PI3K pathway, which affects autoimmunity and causes adverse immune reactions. As shown in Fig. 7, the left part is the domain structure of the BTK enzyme, in which the PH-domain is mainly involved in B cell signal transduction. In the previous report [37], the G →A mutation in the PH-domain causes BTK structural variation and make it fail to recognize the substrate to complete the signal transduction, hinders the development and maturation of B cells. It ends the progenitor B cell phase of the differentiation stage, resulting in a large decrease in plasma cells and affecting the immune response.

In detail, the gene encoding BTK is mutated to form XLA. The defect of BTK leads to the development of B cells in the development of differentiation. Therefore, patients are basically lack of antibody-producing plasma cells, and are susceptible to each The effects of infectious diseases such as Helicobacter. H. cinaedi, formerly known as Campylobacter, in patients with weak immune function, diseases caused by H. cinaedi include repeated fever, bacteremia, arthritis, osteomyelitis, cellulitis, abdominal abscess and diarrhea [38].

In conclusioin, the literature evidence of cellulitis is consistent with the above hypothesis.

### Future of PD-1/PD-L1 targeted therapy

Although PD-1/PD-L1 targeted therapy has proven the enormous power of cancer immunotherapy, in recent studies, anti-PD-1 drugs have no therapeutic effect on non-Hodgkin’s lymphoma, but have a tendency to deteriorate. PD-1 signaling in a mouse model prevents the proliferation of cancerous T cells, and in these mice, anti-PD-1 treatment can aggravate the disease by re-activating cancer cells for continued proliferation [39]. Although this result is individual specific, it also shows that PD-1 inhibitors are not very safe. Activation of the immune system by PD-1/L1 inhibitors also attacks islet cells, resulting in reduced insulin secretion and type 1 diabetes [40]. Ludin and Zon also found that anti-PD-1 therapy may only ameliorate the proliferation of specific T cell subsets that induce cancer, and that anti-PD-1 treatment reduces the effects of phagocytic cells, thereby accelerating tumors. proliferation [41]. In addition, during the treatment of immune checkpoints, not all patients responded to PD-1 monoclonal antibodies, probably because their tumor immunity was limited. Therefore, a combination method for different targets can improve the effect of tumor treatment. The clinical combination of Ipilimumab (an anti-CTLA-4 monoclonal antibody) and Nivoluma has a better therapeutic effect than the use of one antibody alone, but since many side effects have been reported during the single or combined administration, Includes whole body, skin disease, gastrointestinal tract, lung and endocrine [42]. Immunological checkpoint molecules involved in major immunosuppressive effects will vary in different cancers and individual cases, so study the different immunomodulatory mechanisms of the PD-1/PD-L1 pathway in each cell to determine which ones It is important to use a combination of cloned antibody drugs to better enhance the therapeutic effect. It is important to determine the precise mechanism of the combination drug targeting.

PD-1/PD-L1 is not classified according to tumor type but based on biomarkers [14], PD-L1 expression may be a potential predictor of anti-PD-1/PD-L1 antibody therapy, in order to obtain better predictive therapeutic effects as early as possible, especially for predicting PD-1/PD-L1 targeted therapy. A better predictive system of response effects is capable of finding more accurate biomarkers [5]. Immunization-related adverse events against PD-1 therapeutic antibodies are usually reversible and can be well controlled by immunosuppressive therapy, to develop diagnostic tools to determine the most appropriate treatment goals, and to identify with immunological checkpoints predictive biomarkers that are accurately correlated with clinical response or adverse events, so initial diagnosis and close clinical monitoring are critical to successful response to immune-related adverse events.

However, due to the complexity of cancer immunization, the mechanism is still unknown by which PD-1/PD-L1 targeted therapy improves the survival time of cancer patients, so the molecular and cellular mechanisms that clarify the immunosuppressive function of each immunological checkpoint molecule are helpful.

## Data Availability

The data and materials are available upon request.

## Abbreviations

Abbreviations used in this paper are listed below. ADR: Adverse reaction
BioNLP: Biomedical natural language processing
CDR: Complementarity determining region
CRF: Conditional random fields
DISO: Disorder
GO: Gene ontology
HPO: Human phenotype ontology
ICD: Immune checkpoints blockade
PD: Programmed cell death

## References

[1] Couzin-Frankel J. Cancer immunotherapy. Am Assoc Adv Sci. 2013. 10.1126/science.342.6165.1432 24357284

[2] Pardoll DM (2012). The blockade of immune checkpoints in cancer immunotherapy. Nat Rev Cancer.

[3] Dine J, Gordon R, Shames Y, Kasler MK, Barton-Burke M (2017). Immune checkpoint inhibitors: an innovation in immunotherapy for the treatment and management of patients with cancer. Asia-Pacific J Oncol Nurs.

[4] Sharpe AH, Wherry EJ, Ahmed R, Freeman GJ (2007). The function of programmed cell death 1 and its ligands in regulating autoimmunity and infection. Nat Immunol.

[5] Guan J, Lim KS, Mekhail T, Chang C-C (2017). Programmed death ligand-1 (PD-L1) expression in the programmed death receptor-1 (PD-1)/PD-L1 blockade: a key player against various cancers. Arch Pathol Lab Med.

[6] Tang H, Liang Y, Anders RA, Taube JM, Qiu X, Mulgaonkar A, Liu X, Harrington SM, Guo J, Xin Y, et al.PD-L1 on host cells is essential for PD-L1 blockade-mediated tumor regression. J Clin Inv. 2018; 128(2). 10.1172/JCI96061 PMC5785245 29337303

[7] Blank C, Brown I, Peterson AC, Spiotto M, Iwai Y, Honjo T, Gajewski TF (2004). PD-L1/B7H-1 inhibits the effector phase of tumor rejection by T cell receptor (TCR) transgenic CD8+ T cells. Cancer Res.

[8] Bryan LJ, Gordon LI (2015). Blocking tumor escape in hematologic malignancies: the anti-PD-1 strategy. Blood Rev.

[9] Noguchi T, Ward JP, Gubin MM, Arthur CD, Lee SH, Hundal J, Selby MJ, Graziano RF, Mardis ER, Korman AJ (2017). Temporally distinct PD-L1 expression by tumor and host cells contributes to immune escape. Cancer Immunol Res.

[10] Juneja VR, McGuire KA, Manguso RT, LaFleur MW, Collins N, Haining WN, Freeman GJ, Sharpe AH (2017). PD-L1 on tumor cells is sufficient for immune evasion in immunogenic tumors and inhibits CD8 T cell cytotoxicity. J Exp Med.

[11] Fessas P, Lee H, Ikemizu S, Janowitz T. A molecular and preclinical comparison of the PD-1-targeted T-cell checkpoint inhibitors nivolumab and pembrolizumab. In: Seminars in Oncology. volume 44. Elsevier: 2017. p. 136–40. 10.1053/j.seminoncol.2017.06.002 PMC5612055 28923212

[12] Wishart DS, Knox C, Guo AC, Shrivastava S, Hassanali M, Stothard P, Chang Z, Woolsey J (2006). DrugBank: a comprehensive resource for in silico drug discovery and exploration. Nucleic Acids Res.

[13] Wishart DS, Feunang YD, Guo AC, Lo EJ, Marcu A, Grant JR, Sajed T, Johnson D, Li C, Sayeeda Z (2017). DrugBank 5.0: a major update to the DrugBank database for 2018. Nucleic Acids Res.

[14] Hassel JC, Heinzerling L, Aberle J, Bähr O, Eigentler TK, Grimm M-O, Grünwald V, Leipe J, Reinmuth N, Tietze JK (2017). Combined immune checkpoint blockade (anti-PD-1/anti-CTLA-4): Evaluation and management of adverse drug reactions. Cancer Treat Rev.

[15] Eigentler TK, Hassel JC, Berking C, Aberle J, Bachmann O, Grünwald V, Kähler KC, Loquai C, Reinmuth N, Steins M (2016). Diagnosis, monitoring and management of immune-related adverse drug reactions of anti-PD-1 antibody therapy. Cancer Treatment Rev.

[16] Campillos M, Kuhn M, Gavin A-C, Jensen LJ, Bork P (2008). Drug target identification using side-effect similarity. Science.

[17] Keiser MJ, Setola V, Irwin JJ, Laggner C, Abbas AI, Hufeisen SJ, Jensen NH, Kuijer MB, Matos RC, Tran TB (2009). Predicting new molecular targets for known drugs. Nature.

[18] Fang AC, Liu Y, Lu Y, Cao J, Xia J (2018). A corpus-oriented perspective on terminologies of side effect and adverse reaction in support of text retrieval for drug repurposing. Int J Data Min Bioinforma.

[19] Zhou K, Zhang S, Meng X, Luo Q, Wang Y, Ding K, Feng Y, Chen M, Cohen K, Xia J. CRF-LSTM text mining method unveiling the pharmacological mechanism of off-target side effect of anti-multiple myeloma drugs. In: Proceedings of the BioNLP 2018 Workshop: 2018. p. 166–71.

[20] Zhou K, Zhang X, Zweigenbaum P, Liang R, Jiang Y, Xia J. Adverse reaction identification driven by semantic information. In: TAC: 2017.

[21] Wei C-H, Allot A, Leaman R, Lu Z. Pubtator central: automated concept annotation for biomedical full text articles. Nucleic Acids Res. 2019; 47(W1). 10.1093/nar/gkz389 PMC6602571 31114887

[22] Gachloo M, Wang Y, Xia J. A review of drug knowledge discovery using bionlp and tensor or matrix decomposition. Genomics Inf. 2019; 17(2). 10.5808/GI.2019.17.2.e18 PMC6808632 31307133

[23] Cohen KB, Xia J, Roeder C, Hunter LE. Reproducibility in natural language processing: a case study of two r libraries for mining pubmed/medline. In: LREC... International Conference on Language Resources & Evaluation:[proceedings]. International Conference on Language Resources and Evaluation. volume 2016. NIH Public Access: 2016. p. 6. PMC5860830 29568821

[24] Wei Q, Chen T, Xu R, He Y, Gui L. Disease named entity recognition by combining conditional random fields and bidirectional recurrent neural networks. Database. 2016; 2016. 10.1093/database/baw140 PMC5088735 27777244

[25] Liu K, Tan S, Chai Y, Chen D, Song H, Zhang WH, Shi Y, Liu J, Tan W, Lyu J (2017). Structural basis of anti-PD-l1 monoclonal antibody avelumab for tumor therapy. Cell Res.

[26] Lee HT, Ju YL, Lim H, Sang HL, Yu JM, Pyo HJ, Ryu SE, Shin W, Heo YS (2017). Molecular mechanism of PD-1/PD-l1 blockade via anti-PD-l1 antibodies atezolizumab and durvalumab. Sci Rep.

[27] Tan S, Zhang H, Chai Y, Song H, Tong Z, Wang Q, Qi J, Wong G, Zhu X, Liu WJ (2017). An unexpected n-terminal loop in PD-1 dominates binding by nivolumab. Nat Commun.

[28] Roberts K, Demner-Fushman D, Tonning JM. Overview of the TAC 2017 adverse reaction extraction from drug labels track. In: TAC: 2017.

[29] de Leon J (2011). Highlights of drug package inserts and the website DailyMed: the need for further improvement in package inserts to help busy prescribers. J Clin Psychopharmacol.

[30] Köhler S, Vasilevsky NA, Engelstad M, Foster E, Mcmurry J, Aymé S, Baynam G, Bello SM, Boerkoel CF, Boycott KM (2017). The Human Phenotype Ontology in 2017. Nucleic Acids Res.

[31] Korf I, Yandell M, Bedell J. Blast: O’Reilly Media, Inc.; 2003.

[32] Boutet E, Lieberherr D, Tognolli M, Schneider M, Bairoch A. Uniprotkb/swiss-prot. In: Plant Bioinformatics. Springer: 2007. p. 89–112.

[33] Postow MA (2015). Managing immune checkpoint-blocking antibody side effects. Am Soc Clin Oncol Educ Book.

[34] Lavergne T, Cappé O, Yvon F (2010). Practical very large scale CRFs. Proceedings of the 48th Annual Meeting of the Association for Computational Linguistics.

[35] McCray AT, Burgun A, Bodenreider O (2001). Aggregating UMLS semantic types for reducing conceptual complexity. Stud Health Technol Inform.

[36] Bodenreider O (2004). The Unified Medical Language System (UMLS): integrating biomedical terminology. Nucleic Acids Res.

[37] Mattsson PT, Vihinen M, Smith CI. Bioessays News Rev Mol Cell Dev Biol. 2010; 18(10):825–34. 10.1002/bies.950181009 8885720

[38] Sugimoto M, Takeichi T, Muramatsu H, Kojima D, Osada Y, Kono M, Kojima S, Akiyama M (2016). Recurrent cellulitis caused by helicobacter cinaedi in a patient with X-linked agammaglobulinaemia. Acta Dermato-Venereologica.

[39] Wartewig T, Kurgyis Z, Keppler S, Pechloff K, Hameister E, Öllinger R, Maresch R, Buch T, Steiger K, Winter C. Erratum: PD-1 is a haploinsufficient suppressor of T cell lymphomagenesis. Nature. 2017; 553(7683). 10.1038/nature24649 PMC5821214 29143824

[40] Ben NM, Tezza S, D’Addio F, Mameli C, Usuelli V, Maestroni A, Corradi D, Belletti S, Albarello L, Becchi G (2017). PD-l1 genetic overexpression or pharmacological restoration in hematopoietic stem and progenitor cells reverses autoimmune diabetes. Sci Trans Med.

[41] Ludin A, Zon LI. Cancer immunotherapy: The dark side of PD-1 receptor inhibition. Nature. 2017; 552(7683). 10.1038/nature24759 PMC6136420 29143822

[42] Sasidharan NV, Elkord E (2018). Immune checkpoint inhibitors in cancer therapy: a focus on T-regulatory cells. Immunol Cell Biol.

